# Assessment of Circulating Protein Signatures for Kidney Transplantation in Pediatric Recipients

**DOI:** 10.3389/fmed.2017.00080

**Published:** 2017-06-16

**Authors:** Tara K. Sigdel, Minnie M. Sarwal

**Affiliations:** ^1^University of California, San Francisco, San Francisco, CA, United States

**Keywords:** kidney transplantation, serum proteins, transplant injury, acute rejection, protein biomarkers

## Abstract

Identification and use of non-invasive biomarkers for kidney transplantation monitoring is an unmet need. A total of 121 biobanked sera collected from 111 unique kidney transplant (KT) patients (children and adolescent) and 10 age-matched healthy normal controls were used to profile serum proteins using semi-quantitative proteomics. The proteomics data were analyzed to identify panels of serum proteins that were specific to various transplant injuries, which included acute rejection (AR), BK virus nephropathy (BKVN), and chronic allograft nephropathy (CAN). Gene expression data from matching peripheral blood mononuclear cells were interrogated to investigate the association between soluble serum proteins and altered gene expression of corresponding genes in different injury phenotypes. Analysis of the proteomics data identified from different patient phenotypes, with criteria of false discovery rate <0.05 and at least twofold changes in either direction, resulted in a list of 10 proteins that distinguished KT injury from no injury. Similar analyses to identify proteins specific to chronic injury, acute injury, and AR after kidney transplantation identified 22, 6, and 10 proteins, respectively. Elastic-Net logistic regression method was applied on the 137 serum proteins to classify different transplant injuries. This algorithm has identified panels of 10 serum proteins specific for AR, BKVN, and CAN with classification rates 93, 93, and 95%, respectively. The identified proteins could prove to be potential surrogate biomarkers for routine monitoring of the injury status of pediatric KT patients.

## Introduction

Organ transplantation is the treatment of choice for patients with organ failure (1). The number of individuals in the waiting list outnumbers the organs that are available for transplantation (2). Recent advancements in immunosuppressive therapy and surgical techniques have contributed in lowering early acute rejection (AR) events; however, long-term outcome of the transplanted organs is still not satisfactory (3). Since transplanted organs continuously endure injuries that are immune and non-immune related, it is important to reduce such injuries. A better understanding of the detailed mechanism of transplant injury would help in the proper management of transplanted organs for long-term survival (4).

Recent developments in high-throughput assays such as gene microarrays and mass spectrometry-based proteomics have proven to be useful in profiling gene transcripts (mRNA) and proteins, and this global-scale molecular profiling has helped in a better understanding of homeostasis of organ failure (5–9). By analyzing carefully selected samples using advanced statistics, gene microarrays, and mass spectrometry-based proteomics, we can provide a global picture of gene expression and corresponding protein levels specific to disease conditions (6–8, 10–15). We and other groups have previously reported genes, proteins, and antibodies that are associated with different transplant injuries, including AR (5, 10, 12, 16, 17). Our recent studies have demonstrated that mechanisms of graft rejection are common across different solid organs (10, 18, 19). These observations have suggested a strong signal for T cell activation, T cell receptor engagement, and interferon gamma- and STAT1-regulated pathways driven through various chemokines (10). Additional molecular data from carefully selected patient cohorts would provide additional information to our current understanding and provide potential biomarkers for detection of transplant injuries.

The objective of this study was to identify signatures of serum proteins that are associated with KT injury in pediatric patient population. To meet the objective, we used a unique set of 121 serum samples from pediatric patients with kidney transplantation and performed mass spectrometry-based proteomics, followed by statistical and bioinformatic analyses to identify proteins that are associated with different kinds of transplant injuries in kidney. In addition, we also interrogated gene expression data from matching peripheral blood mononuclear cells (PBMCs) to investigate if there existed any correlation between the level of serum proteins and the gene expression of corresponding proteins in matching PBMCs.

## Materials and Methods

### Patient Samples

**Figure 1:**
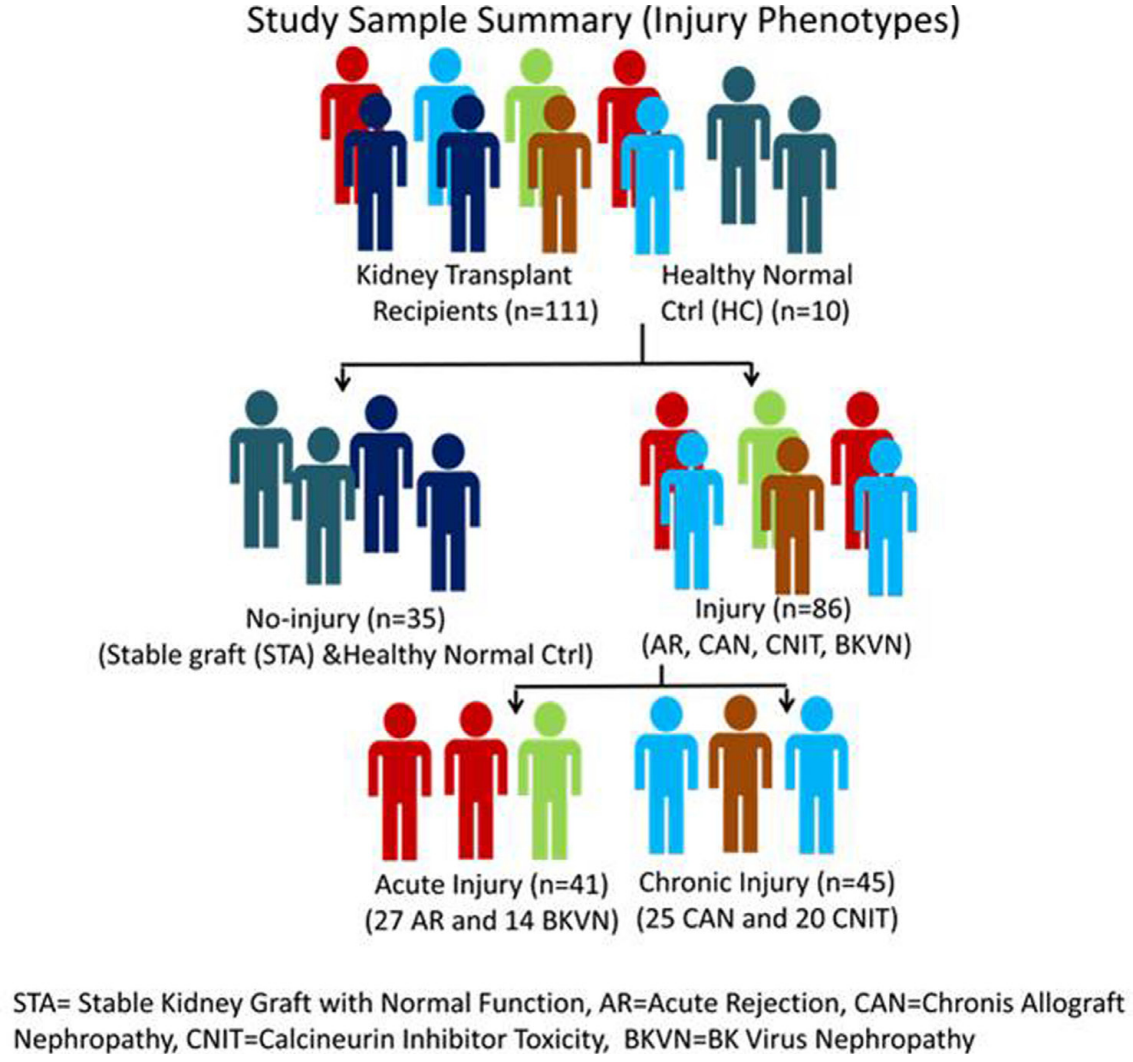
Study sample classification scheme. STA, stable kidney graft with normal function; AR, acute rejection; CAN, chronic allograft nephropathy; CNIT, calcineurin inhibitor toxicity; BKVN, BK virus nephropathy.

Serum samples were collected from 111 unique pediatric kidney transplant (KT) patients from Lucile Packard Children’s Hospital, Stanford University. Sera from 10 age-matched healthy normal controls were also included in the study as a non-transplant control. Each sample was matched with a biopsy collected at the time of serum collection. The study included 27 AR, 20 calcineurin inhibitor toxicity (CNIT), 25 chronic allograft nephropathy (CAN), 14 BK virus nephropathy (BKVN), and 25 normal graft function without significant injury [stable graft function (STA)] samples. All the study samples were collected from pediatric and young adult recipients transplanted between years 2000 and 2011 at Lucile Packard Children’s Hospital of Stanford University. The study was approved by the ethics committees of Stanford University Medical School and UCSF Medical Center. All adult patients and parents/guardians of non-adult patients provided written informed consent to participate in the research, in full adherence to the Declaration of Helsinki. The clinical and research activities being reported are consistent with the Principles of the Declaration of Istanbul as outlined in the Declaration of Istanbul on Organ Trafficking and Transplant Tourism. A summary of demographic information of KT patients and a summary of study samples and analysis scheme are provided in Table 1 and Figure 1. All the kidney biopsies were blindly analyzed by a Stanford University pathologist and were graded by the Banff classification (20–22) for AR, and intragraft C4d stains were performed (23, 24) to assess for acute humoral rejection (25, 26). Transplant “injury” was defined as a >20% increase in serum creatinine from its previous steady-state baseline value and an associated biopsy that was classified as AR, CAN, CNIT, BKVN, or STA. AR was defined at minimum, as per Banff schema, as a tubulitis score ≥1 accompanied with an interstitial inflammation score ≥1 with both C4d and DSA negative. For this study, we included only T-cell-mediated AR. Antibody-mediated rejection cases were excluded from the study. CAN was defined at minimum as a tubular atrophy score ≥1 accompanied by an interstitial fibrosis score ≥1. The histological lesions of chronic CAN were extensively identified, and a semi-quantitative score for CAN was applied to each biopsy, based on standardized definitions from the Banff (2), chronic allograft damage index (3), and chronic CNIT (19) scores. BKVN was defined as positivity of polyomavirus PCR in peripheral blood, together with a positive SV40 stain in the concomitant renal allograft biopsy. Normal (STA) allografts were defined by an absence of significant injury pathology as defined by Banff schema.

**Table 1:** Demographic data of kidney transplant patients used in the study.

Phenotype	AR	STA	CAN	CNIT	BKVN
Number of patients	27	25	25	20	14

Steroid-free/steroid-based	14/13	13/12	11/13	13/7	6/8

Recipient gender (M/F)	18/9	17/8	14/11	13/7	5/7

Recipient race: 1 = White,	1 = 12	1 = 11	1 = 12	1 = 9	1 = 6
2 = Asian,	2 = 6	2 = 7	2 = 7	2 = 5	2 = 2
3 = African American,	3 = 3	3 = 2	3 = 2	3 = 3	3 = 2
4 = Native American and Pacific Islanders,	4 = 1	4 = 1	4 = 2	4 = 0	4 = 0
5 = Mixed and others	5 = 5	5 = 4	5 = 5	5 = 3	5 = 4

Recipient age^a^ (years)	12 ± 5 (14; 10–19)	16 ± 3 (16; 10–19)	12 ± 6 (9; 8–18)	11 ± 6 (11; 3–17)	11 ± 5 (9; 8–18)

Living/deceased	16/11	7/18	16/9	12/8	8/6

Donor gender (M/F)	13/14	14/11	14/11	10/10	9/5

Donor age^a^	28 ± 8 (29; 17–37)	28 ± 10 (27; 14–47)	24 ± 8 (25; 16–31)	28 ± 10 (28; 17–37)	24 ± 8 (25; 16–31)

Average post-Txp time (months)	7 ± 5	6 ± 3	8 ± 4	8 ± 3	8 ± 5

*AR, acute rejection; STA, stable graft function; CAN, chronic allograft nephropathy; CNIT, calcineurin inhibitor toxicity; BKVN, BK virus nephropathy*.

*^a^Age in years: mean ± SD (median; range)*.

### Serum Sample Collection and Storage

Blood samples (4.5 mL) were collected into a 5 mL red top tube and incubated at room temperature for 30 min until the clot was formed. The sample was then centrifuged at 2,000 × *g* for 5 min using a swinging bucket rotor. The upper layer of serum was then transferred to another cryotube and was stored at −80°C until use.

### Mass Spectrometry

Serum samples were depleted of the 20 most abundant serum proteins by ProteoPrep20 Plasma Immunodepletion Kit (Sigma-Aldrich, St. Louis, MO, USA, Cat. PROT20). The eluate from each sample was subsequently subjected to trypsin digestion with a standard trypsin digestion protocol. Tryptic peptides were reconstituted in a buffer containing 0.2% formic acid, 2% acetonitrile, and 97.8% water prior to mass spectrometry. The high-performance liquid chromatography utilized was an Eksigent nano2D (Eksigent) with a self-packed 150 μM ID C18, 15 cm column. The electrospray source was a Michrom Advance operated at 600 nL/min on an LTQ Orbitrap Velos (Thermo Fisher). Data acquisition was performed in a data-dependent manner in which the top 12 (Velos) most intense-charged peptide ions were selected for MS/MS fragmentation of charge state 2+ and 3+. Data were subsequently extracted with msconvert script into an mzXML format prior to Sorcerer (SAGE-N) analysis with the Sequest algorithm. The IPI human database was searched using a 50 ppm mass window on the precursor ion. We allowed for the static modification of propionamide on cysteine and variable modifications of methionine oxidation and lysine acetylation. All searches were compiled and displayed in a Scaffold (Proteome Software Inc., Portland, OR, USA) interface, which listed the identified proteins with cumulative spectral counts for each protein.

### Gene Expression Analysis

Gene expression analysis was performed for gene expression aberrations on a subset of whole blood collected at the same time from the study subjects for the proteomics study. This analysis included blood samples collected from 11 AR, 11 STA, 9 CAN, 9 BKVN, and 9 CNIT patients. Blood was collected in 2.5 mL PAXgene™ Blood RNA Tubes (PreAnalytiX, Qiagen, Valencia, CA, USA). Total RNA was extracted, and cDNA synthesis was done using a previously published protocol (27). Synthesized cDNA was then hybridized onto GeneChip Human Genome U133 Plus 2.0 Arrays (Affymetrix Inc., Santa Clara, CA, USA). The arrays were washed and scanned as recommended by the Ovation Biotin RNA Amplification and Labeling System User Guide (version 1.0) (NuGEN Inc., San Carlos, CA, USA). The data were analyzed using AltAnalyze software (28). Genes were considered to have differentially expressed by an empirical Bayes *t* test, *p* value <0.05, and fold change 1.5.

### Data Analysis

Data analysis was done in three steps. First, we performed ANOVA to identify injury-specific serum proteins in KT injury. In this step, mass spectrometric data were analyzed with settings that included the false discovery rate (FDR) for protein identifications 0.1% based on target decoy analysis using Sequest^®^ and the FDR at the unique peptide level 0.2%. The spectral counts for the identical IPI numbers were summed. After filtering for proteins that were not consistently identified in different samples, the identified proteins were used for further analysis. In the first step, the data were quantile-normalized and used for ANOVA analysis. Phenotype-specific proteins were identified with ANOVA with the criteria FDR <0.05 and at least twofold change in either direction. Principal component analysis (PCA) plots and heatmaps were generated using Partek Genomics Suite (Partek Inc., St. Louis, MO, USA).

In the second step, we used penalized logistic regression to identify proteins that could serve as potential surrogate biomarkers for different KT injuries. This approach provides not only accurate estimation for the regression coefficients but also probability estimation for each patient (29, 30). We used the regularization paths for generalized linear models *via* Coordinate Descent for the estimations (30). The logistic equation used is:

$$\text{log-odds}=\text{ln}\left(\frac{\pi (x)}{1-\pi (x)}\right)=\beta_{0}+\beta_{1}x_{1}+\beta_{2}x_{2}+\cdots +\beta_{K}x_{K}$$

where π(*x*) = *P*(*x*) (*case*) and *x*1, …, *x_K_* are the expressions of *K* proteins for observations *x*. The Elastic-Net fits this model by adding a mixed penalty term to the likelihood

$$l(\beta |x)=-\overset{n}{\underset{i=1}{\sum }}\text{log}(1+\text{exp}(-y_{i}\beta^{T}x))-\lambda \left(\alpha \overset{K}{\underset{k=1}{\sum }}\beta_{k}^{2}+(1-\alpha )\overset{K}{\underset{k=1}{\sum }}\left|\beta_{k}\right|\right).$$

We fitted 100 Elastic-Net logistic regression models to the 137 proteins using bootstrapped samples maintaining the use of ~75% samples in the training set and ~25% samples in the test set to classify between transplant samples and non-transplant samples with different transplant injury status. For each bootstrap, a nested cross-validation loop estimated the best value according to the deviance. The parameter of the Elastic-Net was fixed at 0.95, the value recommended by Friedman et al. (30). The mean test classification rate was 96%. We counted the number of times each protein was selected by the Elastic-Net over the 100 bootstraps. For each of the bootstrap samples, the Elastic-Net fit a subset of the 137 proteins with non-zero coefficients. For each protein, we counted the number of bootstrap samples for which the protein had non-zero coefficients. After running the 100 bootstrapped models, we selected the K proteins with the greatest number of non-zero coefficients. Classification with the reduced set of K proteins: in order to have an unbiased estimation of the predictive performance (classification rate, sensitivity, specificity, PPV, and NPV), we ran another set of 100 bootstrap Elastic-Net classifications with nested cross-validation for λ, this time using only the set of K proteins selected in step 1. Finally, in the third step, we fitted the selected models to the whole dataset to give the regression coefficients.

## Results

The objective of this study was to identify KT injury-specific proteins. By the proteomics analysis of serum proteins, we identified 137 proteins across all the samples with the criteria of (i) minimum two peptides per protein for a positive identification and (ii) 0.1% FDR for protein and 0.2% FDR for peptide identification Sequest^®^ (31). An enrichment analysis for the biological association of the 137 proteins listed complement and coagulation cascade (*p* = 4.79E−38) and peptidase inhibitor activity (*p* = 1.02E−13) as the top associations of the proteins identified.

### Serum Proteins Specific to Different Injury Subtypes

**Figure 2:**
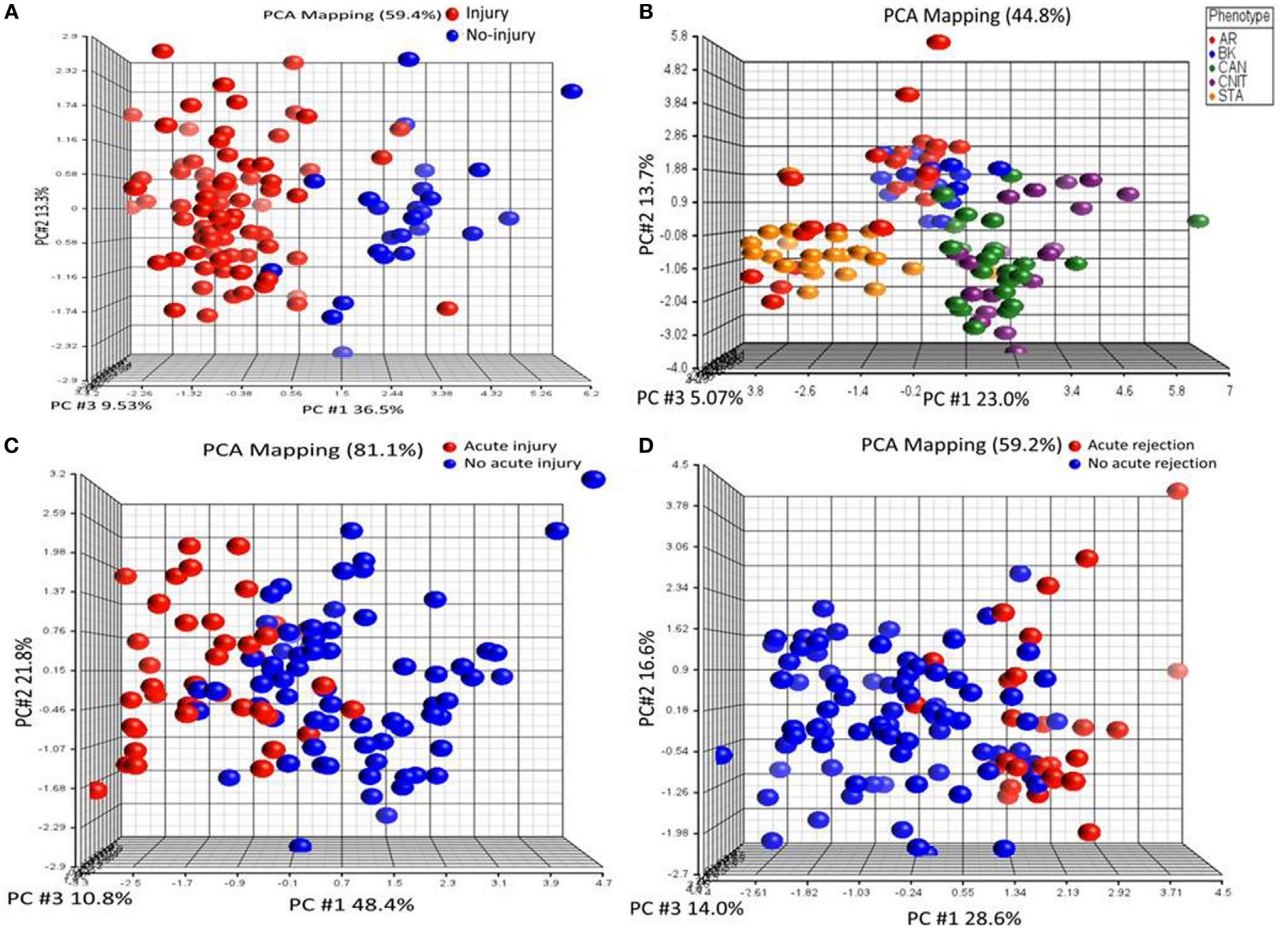
Serum proteins specific to different transplant injury phenotypes were identified. **(A)** A principal component analysis (PCA) plot generated by 10 serum proteins that separate samples collected from patients with kidney injury from samples collected from patients with no kidney injury. **(B)** A PCA plot generated by 22 serum proteins that separate chronic injury samples from samples collected from patients with no chronic injury in their kidney. **(C)** A PCA plot generated by six serum proteins that separate serum samples from the patients with acute injury from the samples collected from patients with no acute injury. **(D)** A PCA plot generated by 10 serum proteins that separate serum samples from the patients with acute rejection (AR) from the samples collected from patients with no AR.

To identify KT injury status, ANOVA was performed on proteomics data with the criteria of FDR <0.05 and at least twofold change in either direction for significance. (i) *Injury vs no injury*: for this, the samples were classified in to injury (AR, CAN, CNIT, BKVN) and no injury (STA and HC). Ten differentially expressed unique proteins were identified specific to kidney injury after transplantation (Table 2). These 10 proteins were significantly associated with a complement activation pathway (FDR = 0.006). (ii) *Chronic injury-specific serum protein markers*: the contrast between CAN/CNIT (Chronic) and AR/BK/STA (non-chronic) identified 22 differentially expressed proteins listed in Table 2. These proteins are associated with positive regulation of cholesterol esterification (FDR = 5.26E−10), plasma lipoprotein particle remodeling (FDR = 5.64E−08), regulation of response to wounding (FDR = 5.64E−08), and regulation of inflammatory response (FDR = 1.02E−07). (iii) *Acute injury-specific proteins*: we identified 6 differentially expressed proteins between patients with acute injury (AR and BKVN) and those with chronic injury (CAN and CNIT) or STA (Table 2). These proteins are associated with blood coagulation pathways. (iv) *Identification of AR with 10 proteins*: 10 differentially expressed proteins were identified between patients with AR and those without AR (CAN, CNIT, BKVN, and STA) (Table 2). These 10 proteins are associated with defense response (FDR = 0.006), positive regulation of cholesterol esterification (FDR = 0.005), and positive regulation of triglyceride catabolic process (FDR = 0.006). PCA on the proteins identified for injury, chronic injury, acute injury, and AR demonstrated a good separation of the phenotypes (Figures 2A–D).

**Table 2:** List of serum proteins specific to kidney transplant injury.

Transplant vs no transplant (acute rejection (AR), chronic allograft nephropathy, calcineurin inhibitor toxicity, BK virus nephropathy, stable graft function vs HC)			

S. no.	Protein name	*p*-Value (injury vs no injury)	Ratio (injury vs no injury)
1	Complement factor properdin (CFP)	8.85E−06	8.47E−03
2	CXCL7	2.73E−05	6.01E−04
3	Afamin (AFM)	6.27E−05	2.73E−09
4	IGHG4 immunoglobulin heavy constant gamma 4	3.89E−04	1.03E−03
5	Clusterin	1.19E−03	7.62E−04
6	Apolipoprotein A-II (APOA2)	1.51E−03	1.31E−03
7	Uncharacterized protein	3.76E−03	9.37E−04
8	Butyrylcholinesterase (BCHE)	2.91E−04	9.45E+01
9	Lumican (LUM)	3.09E−04	2.02E+03
10	C8G complement component 8, gamma polypeptide	2.63E−03	4.06E+01

**Chronic injury**			

	**Protein name**	***p*-Value (chronic injury vs non-chronic)**	**Ratio (chronic injury vs non-chronic)**

1	Apolipoprotein A-IV (APOA4)	5.19E−16	5.16E−14
2	Fibronectin 1 (FN1)	5.90E−05	9.40E−09
3	CFP	4.40E−04	1.12E−01
4	AFM	2.26E−03	1.59E−04
5	PROS1 protein S	2.84E−03	1.91E−01
6	AGT angiotensinogen	2.94E−03	2.51E−02
7	Complement factor B (CFB)	4.62E−03	2.39E−05
8	C5 complement component 5	4.82E−03	6.99E−04
9	Apolipoprotein E	5.62E−03	7.46E−03
10	LUM	8.42E−03	2.54E+01
11	Attractin (ATRN)	8.22E−13	2.88E+02
12	alpha-2-HS-glycoprotein (AHSG)	1.57E−10	1.61E+11
13	Histidine rich glycoprotein (HRG)	3.89E−07	7.89E+02
14	BCHE	3.43E−05	2.23E+01
15	IGHV4-31	1.09E−04	3.90E+06
16	CPN2, Carboxypeptidase N	1.67E−03	1.24E+01
17	A1BG alpha-1-B glycoprotein	2.69E−03	4.14E+04
18	IGHM	2.73E−03	2.52E+03
19	Apolipoprotein C-I	2.84E−03	1.81E+01
20	Apolipoprotein A-I (APOA1)	2.88E−03	4.23E+22
21	SELL selectin L	2.96E−03	4.91E+00
22	LGALS3BP lectin galactoside-binding, etc.	3.63E−03	1.11E+01

**Acute injury**			

	**Protein name**	***p*-Value (acute vs non)**	**Ratio (acute vs non)**

1	AHSG	5.59E−14	2.06E−15
2	ATRN	4.84E−11	4.26E−03
3	IGHV4-31	5.94E−04	6.09E−07
4	IGHG2	1.16E−03	5.24E−05
5	APOA4	2.83E−11	1.11E+11
6	FN1	2.17E−03	2.84E+06

**AR**			

	**Protein name**	***p*-Value (AR vs no AR)**	**Ratio (AR vs no AR)**

1	AHSG	1.68E−08	3.71E−11
2	APOA4	1.10E−06	5.49E+07
3	ATRN	2.09E−05	3.54E−02
4	CFB	6.53E−04	1.15E+06
5	SERPINA3	7.04E−04	2.07E+16
6	APOA1	1.25E−03	2.65E−27
7	IGHG2	1.35E−03	5.53E−05
8	C5 complement component 5	1.70E−03	6.50E+03
9	IGHA1 immunoglobulin heavy constant alpha 1	2.29E−03	1.36E+02
10	HRG	2.47E−03	1.62E−02

### Identification of Serum Protein Panel for Different KT Injuries

**Figure 3:**
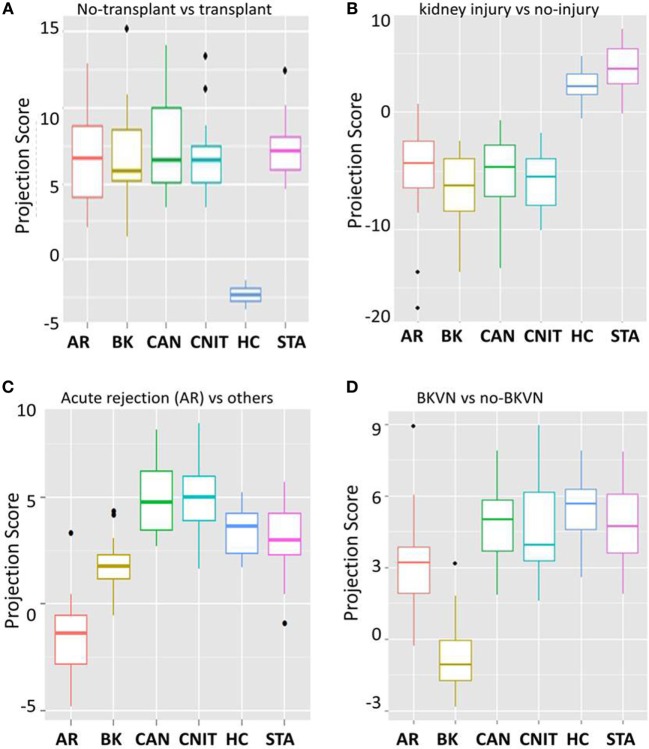
10 protein biomarker models for different kidney injury phenotypes. **(A)** A box-and-whisker plot for 10 protein panel specific for transplantation when compared to no transplant healthy normal controls (HC). **(B)** A box-and-whisker plot for 10 protein panel specific for kidney injury due to kidney transplantation when compared to well-functioning kidneys after transplantation [stable graft function (STA)] and healthy normal controls (HC). **(C)** A box-and-whisker plot for 10 protein panel specific for acute rejection (AR) when compared to no AR phenotypes. **(D)** A box-and-whisker plot for 10 protein panel specific for BK virus nephropathy (BKVN) when compared to serum from patients with absence of BKVN.

To identify transplant injury-specific panels, we fitted 100 Elastic-Net logistic regression models to the 137 proteins using bootstrapped samples maintaining the use of ~75% samples in the training set and ~25% samples in the test set to classify transplant samples and non-transplant samples with different transplant injury status. (i) *KT-specific panel*: analysis of healthy normal control (HC) vs transplant samples identified 10 proteins (APOB, GPLD1, PLG, 66 kDa protein (IPI00941961.1), CP, ALB, apolipoprotein A-II (APOA2), IGHG3, ORM2, and SAA4) on the whole dataset as proteins specific to transplantation. The box-and-whisker plot in Figure 3A shows separation of projection score for different phenotypes. The ROC analysis was performed for the classification potential, which resulted in AUC of one by the model consisting of 10 proteins. The interception and regression coefficients are shown in Table 3. (ii) *Injury-specific protein panel*: next, we sought to identify a panel of proteins that separate serum samples with kidney injury from those with well-functioning kidneys. A panel of 10 proteins was identified, which classified injury (AR, CAN, CNIT, BKVN) (*n* = 86) vs no injury (STA and HC) (*n* = 35). The mean classification rate, sensitivity, specificity, PPV, and NPV were 0.92. 0.96, 0.84, 0.93, and 0.91, respectively. The interception and regression coefficients are shown in Table 3. (iii) *Acute injury protein panel*: next, we identified 10 proteins that were specific to acute transplant injury (AR and BKVN) compared with no acute injury (CAN, CNIT, HC, and STA). The mean classification rate for the 10 protein panel was 98% [CI 95% = (95.8, 100)], sensitivity = 0.95, specificity = 0.98, PPV = 0.95, NPV = 0.98. The interception and regression coefficients are shown in Table 3. (iv) *Biomarker protein panel for AR*: the analysis that compared protein signal from AR samples against all non-AR samples identified 10 proteins to classify AR (*n* = 27) vs others (CAN, CNIT, BKVN, STA, and HC) (*n* = 94). The mean classification rate was 0.91. The sensitivity, specificity, PPV, and NPV were 0.74, 0.96, 0.83, and 0.93, respectively. The interception and regression coefficients are shown in Table 3. Figure 3C shows the separation of projection score for AR with other phenotypes. (v) *Biomarker proteins for BKVN*: a panel of 10 proteins was identified that was able to classify BKVN (*n* = 14) vs others (*n* = 107) with a mean classification rate 0.93. The sensitivity, specificity, PPV, and NPV were 0.65, 0.96, 0.69, and 0.95, respectively. The interception and regression coefficients are shown in Table 3. Figure 3 shows the separation of projection score for AR with other phenotypes. (vi) *Serum protein biomarkers to distinguish CAN vs CNIT*: a panel of 10 proteins was identified that classified CAN (*n* = 25) vs CNIT (*n* = 20). The mean classification rate was 0.95. The sensitivity, specificity, PPV, and NPV were 0.96, 0.94, 0.96, and 0.95, respectively. The interception and regression coefficients are shown in Table 3.

**Table 3:** Serum protein panels for kidney transplant injury by penalized logistic regression analysis.

Description		Intercept	Protein 1	Protein 2	Protein 3	Protein 4	Protein 5	Protein 6	Protein 7	Protein 8	Protein 9	Protein 10
Non-transplant vs transplant	10 protein model		APOB	GPLD1	PLG	66 kDa protein	CP	ALB	Apolipoprotein A-II	IGHG3	ORM2	SAA4
	Regression coefficients	29.71	−2.46	−0.56	−0.39	−1.07	0.22	−0.43	0.25	0.25	0.01	0.00

Kidney injury vs no injury	10 protein model		HBB	Lumican (LUM)	Complement factor properdin	LGALS3BP	C1R	SERPINA4	HBD	APOB	PPBP	IGHG4
	Regression coefficients	−1.23	−0.95	−1.35	0.41	−0.71	−0.70	0.99	−0.379	0.82	0.67	0.22

Acute injury vs no acute injury	10 protein model		Apolipoprotein A-I (APOA1)	HBB	Apolipoprotein A-IV (APOA4)	Attractin (ATRN)	VWF	FETUB	LUM	VTN	IGK alpha	AZGP1
	Regression coefficients	0.24	2.35	−1.01	−1.01	1.11	−0.30	−0.82	−1.38	−2.16	1.33	0.52

Acute rejection (AR) vs no AR	10 protein model		APOA1	VWF	ECM1	Alpha-2-HS-glycoprotein	ATRN	FETUB	Histidine rich glycoprotein	PF4	AZGP1	ORM1
	Regression coefficients	−4.55	0.67	−0.39	−0.53	0.40	0.45	−0.40	0.92	−0.43	0.32	−0.76

BK virus nephropathy vs others	10 protein model		LPA	APOA4	IGHA2	PF4	HBB	SERPINF1	PPBP	ATRN	HBA2HBA1	Afamin
	Regression coefficients	2.57	−0.50	−0.39	0.25	0.65	−0.006	0.67	0.29	0.00	−0.29	0.108

Chronic allograft nephropathy vs calcineurin inhibitor toxicity	10 protein model		IGHA1	TTR	LUM	GC.1	IGHG2	SERPINA4	APOL1	F12	FN1	CPN1
	Regression coefficients	−11.09	2.03	1.25	4.17	1.45	1.37	−2.27	1.10	−2.01	−1.02	−0.45

### Gene Expression Analysis of Whole Blood from a Subset of Serum Samples Used in Proteomics Analysis

A subset of PBMCs with matching serum samples from the same collection date for each patient was analyzed. A total of 9,665 genes were significantly different in whole blood by ANOVA (*p* < 0.05). Among these, only 39 proteins (gene products) were identified in our serum proteomics data. With the significance criteria of an empirical Bayes *t* test *p* value <0.05 and a fold change of two vs stable graft (STA), a total of 626 upregulated genes were identified, which enriched for the biological system process (FDR = 0.0004). In addition, there were 7,316 downregulated genes enriched in AR, which are associated with cellular macromolecule metabolic process (FDR = 5.1E−14) and primary metabolic process (FDR = 2.1E−13). There were 12 upregulated and 713 downregulated genes in CAN. CAN-associated genes were highly enriched in immune system process (FDR = 7.4E−9) and response to stress (FDR = 2.4E−6). Blood from BKVN patients demonstrated 141 upregulated and 1,321 downregulated genes in BKVN, which were enriched in anatomical structure morphogenesis (FDR = 4.2E−05) and transcription regulatory region DNA binding (FDR = 0.005). There were seven upregulated and 290 downregulated genes associated with CNIT. These CNIT-associated genes were enriched in viral transcription process (FDR = 0.003). Even though we were not expecting to see correlation between serum protein level and gene expression in the whole blood, we noticed that some serum protein levels had aligned with corresponding gene expression levels in the PBMCs. Among those genes, complement factor properdin (CFP), attractin (ATRN), serum IgA (IGHG2 – gene), and clusterin (CLU) levels were downregulated in acute injury, including AR (both gene expression and protein level).

## Discussion

Despite the fact that there is an urgent need to improve long-term survival of transplanted organs, the organs succumb to immune- and non-immune-related causes (32). Sequencing of human genome and subsequent development of “omic” platforms have led us to investigate the biology of transplant rejection and failure (15, 17, 33). The data presented in recently published reports have helped our understanding in immune-related processes (3–7). Similar studies to identify serum proteins in transplant injuries have been published, but they suffer from either small sample size (34) or a lack of diversity of injuries to really understand the perturbations of those proteins (35). In addition, rejection is a heterogenous biological process, and it has been accepted that the possibility that a single gene or protein could serve as a surrogate biomarker or represent the multitude of biological events that occur concurrently during transplant injury (36) is unlikely. The semi-quantitative proteomics strategy used in this study and the penalized logistic regression to identify potential biomarker protein panels for different KT injury phenotypes is a novel approach that we used in the context of biomarker discovery of serum proteins in kidney transplantation. The method has provided accurate estimates for the regression coefficients and probability estimates for each transplant injury phenotype in pediatric KT recipients (29, 30).

There are several important findings in this report. First, we identified proteins associated with different transplant injuries. A broad analysis to identify transplant injury-associated proteins listed proteins such as CFP, which is known as a positive regulator of complement activation (37), and CLU, which is known to help with the clearance of cellular debris and apoptosis (38). Six out of eight proteins identified were associated with complement activation and alternative pathways. Complement and coagulation cascade-related proteins such as complement component (C5), complement factor B (CFB), and Protein S alpha (PROS1) were associated with chronic injury. Involvement of the complement system in transplant rejection and chronic injury has been reported (39, 40). Association of complement and coagulation cascade-related proteins is relevant as a response to immune system and vascular injury to the organ. A set of immune regulatory proteins namely alpha-2-HS-glycoprotein (AHSG), apolipoprotein A-IV (APOA4), ATRN, IgG, and fibronectin 1 (FN1) were associated with acute injury. Fibronectin is known to participate in cell adhesion, growth, migration, and differentiation (41). Soluble FN is expressed in hepatocytes (42) and is produced as a response to vascular injury (43). Our observation of a decreased level of serum fibronectin (FN) in chronic injury and increased level of FN in acute injury, which includes AR and BKVN, is interesting as its protein level in in the blood and expression in the rat kidney has been studied in the context of transplant rejection with no clear association (44–47). Molecules involved in the regulation of defense and inflammatory response are namely AHSG/fetuin-A, APOA4, ATRN, CFB, SERPINA3, apolipoprotein A-I, IgG, complement component 5 (C5), immunoglobulin heavy constant alpha 1 (IgA), and histidine-rich glycoprotein. The elevation of these proteins in the blood during an AR episode is attributed to the response to the acute inflammation due to rejection.

Second, using a penalized logistic regression panel, we have identified panels of 10 proteins as a surrogate biomarker panel for different transplant injuries. After several efforts in biomarker discovery for AR and other transplant injuries, it has now become evident that finding a single gene or protein as a biomarker is almost impossible. Biomarker discovery and validation is an arduous task and takes a long-term study and many validation steps. Even though we started off with identifying the panel of potential biomarker proteins, and validation of biomarker panels was not the scope of this study, we utilized data generated in this to identify potential biomarker panels for transplant injuries and validated them using ~25% of the study samples. To this end, using our penalized logistic regression model, we have identified 10 protein panels for transplant, transplant injury, AR, acute injury that included AR and BKVN, and a panel that differentiates CAN from CNIT. The sensitivity, specificity, PPV, and NPV are listed for different models, which show some promise for these proteins to serve as biomarkers. We acknowledge the small sample size and therefore consequently our results could suffer from overfitting. Validation of this will require use of more prospectively collected samples in a clinical trial setting on a larger cohort of patient samples.

The third important finding of this study is that profiling of serum proteins and profiling of transcriptome from whole blood provided complementary information about the perturbations in the blood in case of other injuries and allowed us to compare complementary information about molecular pathways that were obtained from serum proteomics and gene expression of the blood cells. Even though the overlap in between the serum proteins (*n* = 137) to gene expression data (*n* = ~20,000) was minimal, this is our effort to understand the molecular perturbations in different tissue types. This approach is important as analyzing different tissue types will help build a complete picture of biological events that occur during an immune or non-immune insult to the transplanted kidney.

In conclusion, using a high-throughput semi-quantitative mass spectrometry-based proteomics approach with serum samples from pediatric KT patients; we have identified proteins associated with different transplant injuries in kidney transplantation. We acknowledge some limitations of this study, including that its conclusions are based on discovery data and lack independent validation. We acknowledge that the use of a strategy that used depletion of high abundant proteins, including the possibility that we depleted any potential biomarker that was albumin-bound. Every discovery study like this should be followed up with a larger validation study using an independent cohort of samples for proteins and genes as a surrogate biomarker for KT injury. With this promising result in hand, we are planning studies that will include validation of identified proteins and genes in a set of prospectively collected samples. This longitudinal sample-set will also determine whether the increased or decreased level of the serum proteins correlates with injury status over time.

## Ethics Statement

This study was carried out in accordance with the recommendations of the University of California San Francisco with written informed consent from all subjects. All subjects gave written informed consent in accordance with the Declaration of Helsinki. The protocol was approved by the Human Research Protection Program, Institutional Review Board (IRB) of University of California San Francisco.

## Author Contributions

TS and MS conceptualized the study. TS carried out the experiments and data generation. Both TS and MS analyzed the data and prepared the manuscript.

## Conflict of Interest Statement

The authors declare that the research was conducted in the absence of any commercial or financial relationships that could be construed as a potential conflict of interest.

## Abbreviations

AR, acute rejection; CAN, chronic allograft nephropathy; BKVN, BK virus nephropathy; CNIT, calcineurin inhibitor toxicity; STA, stable graft function.
